# Endoscopic endonasal approach for brainstem cavernous malformation

**DOI:** 10.3171/2019.10.FocusVid.19399

**Published:** 2019-10-01

**Authors:** Ezequiel Goldschmidt, Andrew S. Venteicher, Maximiliano Nuñez, Eric Wang, Carl Snyderman, Paul Gardner

**Affiliations:** 1 Departments of Neurosurgery and; 2 Otolaryngology, University of Pittsburgh Medical Center, Pittsburgh, Pennsylvania

**Keywords:** endonasal endoscopic approach, brainstem, cavernous malformation, video

## Abstract

This 25-year-old woman presented after a second hemorrhage from a mesencephalic cavernous malformation. High-definition fiber tracking demonstrated lateral displacement of the corticospinal tracts, making a midline approach ideal. The lesion appeared to present to the third ventricle, but a transcallosal approach was abandoned due to the posterior third ventricular location and after FIESTA imaging revealed a superior and medial rim of normal parenchyma that would have to be transgressed to access the malformation. An endoscopic endonasal approach with interdural pituitary hemitransposition was performed. The interpeduncular cistern was accessed and the thalamoperforating arteries dissected to access the cavernous malformation that was completely removed in a piecemeal fashion. The patient’s preexisting internuclear ocular palsies and hemiparesis were slightly worsened after surgery as predicted by a drop in anterior tibialis motor evoked potentials. Postoperative MRI showed no infarct, and the hemiparesis was back to baseline at 1-month follow-up.

The video can be found here: https://youtu.be/e6203R9HHmk.

## Transcript

This is the case of an endoscopic endonasal resection of a brainstem cav mal. It is a 25-year-old woman who had two episodes of hemorrhage, one with initial diplopia and headache, then developing ventriculomegaly and hydrocephalus treated with an ETV. She then presented with a clear second hemorrhage and worsening double vision. Here you can see her progression over time of this midbrain malformation. It did not quite present to the floor of the third ventricle; it seemed to present in the midline to the mesencephalon. Careful examination of imaging presented concern for multiple approaches; here we see a FIESTA sagittal where again you can see the central location of the cavernous malformation. High-def fiber tracking was performed to try to understand the relationship of critical tracts. They showed lateral displacement of the motor fibers as well as third nerve tract, which made a ventral medial approach theoretically an ideal approach for this lesion. Endonasal and transcranial options were both considered; however, an interhemispheric transcallosal option was abandoned due to the location in the posterior third ventricle and after review of the sagittal FIESTA imaging revealed a thin rim of viable tissue between the cavernous malformation and the floor of the third ventricle. We therefore elected for an endoscopic endonasal resection. Of course, this requires multiple stages to it, but did require a pituitary hemitransposition on the right side of the pituitary gland in order to access this region of the mesencephalon. Here we can see the region of access just below the mamillary bodies, above the basilar apex. Nasal stage consisted on the resection of the middle turbinate on the right side. A right-sided nasoseptal flap with a left-sided reverse flap, so-called Caisedo flap. Next a wide sphenoidotomy was performed as well as a posterior ethmoidectomy. Here we can see the wide sphenoidotomy. A posterior septectomy is part of the reverse flap, and it helps provide wide view and access. The bone overlying the sella and the tuberculum and planum were carefully thinned and removed. This is done in the usual fashion where rather than biting with a rongeour, the bone is carefully thinned and then pealed away. Here the right parasellar carotid artery is exposed by again carefully blue-lining the bone and then carefully pealing the bone from the cavernous sinus. The upper and midclivus is necessary in order to have access to the basilar apex and have proximal control in the event of a vascular injury. Here we can see the midclivus being opened. Next, for a superior clival access, the cavernous sinus is opened on the right side and packed off with Gelfoam packing, and then the remainder of the medial cavernous sinus is widely opened. This is taken up to the diaphragm, which is also split adjacent to the pituitary stalk. This allows for complete pituitary transposition and mobilization of the gland towards the left side. Here we can see access to the very tall posterior clinoid. Once this has been dissected free, and the inferior hypophyseal artery is sacrificed, the posterior clinoid can be completely resected and the cap of it can be peeled from behind the carotid artery. This then allowed us to open the upper clival dura in addition. We can now see arachnoid being open, the third nerve. We can then dissect back along the third nerve to the basilar apex and then the mamillary bodies come into view. Here, examination of the prior endoscopic third ventriculostomy showed lack of patency. This was dissected and slightly reopened to allow relaxation of the mamillary bodies away from the basilar apex. We can then dissect and evaluate the thalamoperforators, the basilar apex, and also see some vague discoloration in the midbrain where the cavernous malformation essentially presents to the surface. Indocyanine green angiography with the endoscope was performed to evaluate the vasculature, and again here we see the region of the mesencephalon where the cavernous malformation presents to the surface in the anterior midline. We check again for perforators; we confirm our localization. Here you see a dorsal view of the brainstem anatomy related to this cavernous malformation. Midline dissection with microendoscopic forceps is performed and gain us access into the hematoma cavity. We begin to see the lesion and is removed piecemeal with careful visualization, dissection around the edge and grasping of the lesion, and delivery into the surgical field. Each pass is taken carefully with visualization of the thalamoperforators and the mamillary bodies. Image guidance helps confirm depth within the brainstem as we carefully evaluate for complete resection. Persistent bleeding reveals that there is likely residual left, so further exploration is performed for continued piecemeal removal. Dynamic endoscopy is critical for this portion as two suctions and forceps are used to carefully clear the field, evaluate the hematoma cavity, and visualize more malformation. Abnormal fluorescence is evaluated throughout. The window here between the mamillary bodies and the basilar apex is key. Image guidance helps confirm our depth. Obviously in this location in the brainstem image guidance is important for confirmation of location. This midline approach has allowed us to evaluate the cavernous malformation directly, deliver large portions like this without any disruption or transgression of the laterally displaced corticospinal tracts, oculomotor nuclei, and nerves. Careful inspection of the cavity at the end confirms complete resection. Meticulous hemostasis. After meticulous hemostasis repeat ICG angiography confirms patency of the basilar as well as the PCAs and the thalamoperforators. Reconstruction is performed in a multilayered fashion with a collagen matrix inlay graft. This is then followed by a vascularized nasoseptal flap. The large size of the flap compared to the relatively large opening allowed us to only use these two layers for reconstruction. Given the clival exposure we then placed a lumbar drain in this patient. She was discharged five days later. She had a slightly worsened hemiparesis and difficulty with eyelid opening, but that slowly improved over time and is now back to her preoperative baseline. Postoperative imaging shows a complete removal of the cavernous malformation with no new infarct.

## Time points

0:20 Case presentation
1:44 Approach selection
1:44 Surgical strategy
2:05 Nasal stage
2:30 Sphenoid stage
3:00 Clival stage
3:22 Pituitary transposition
3:56 Subarachnoid stage
4:20 Exploration of the third ventriculostomy
4:59 Parenchymal stage and removal of the lesion
7:00 Skull base reconstruction
7:19 Postoperative course

## References

[1] Fernandes Cabral DT Zenonos GA Nuñez M Celtikci P Snyderman C Wang E Endoscopic endonasal transclival approach for resection of a pontine glioma: surgical planning, surgical anatomy, and technique Oper Neurosurg 15 589 599 2018 10.1093/ons/opy005 29538708

[2] Flores BC Whittemore AR Samson DS Barnett SL The utility of preoperative diffusion tensor imaging in the surgical management of brainstem cavernous malformations J Neurosurg 122 653 662 2015 25574568 10.3171/2014.11.JNS13680

[3] Gode S Lieber S Nakassa ACI Wang EW Fernandez-Miranda JC Gardner PA Clinical experience with secondary endoscopic reconstruction of clival defects with extracranial pericranial flaps J Neurol Surg Part B Skull Base 80 276 282 2019 10.1055/s-0038-1668517 PMC6534769 31143571

[4] Goldschmidt E Hem S Ajler P Ielpi M Loresi M Giunta D A new model for dura mater healing: human dural fibroblast culture Neurol Res 35 300 307 2013 23336298 10.1179/1743132812Y.0000000136

[5] Jaman E Goldschmidt E Algattas H Wang E Snyderman CH Gardner PA Hormonal fertility therapy as potential risk factor for cerebrospinal fluid leak after endoscopic endonasal surgery: case study and literature review World Neurosurg 128 458 463 2019 31132487 10.1016/j.wneu.2019.05.134

[6] Januszewski J Albert L Black K Dehdashti AR The usefulness of diffusion tensor imaging and tractography in surgery of brainstem cavernous malformations World Neurosurg 93 377 388 2016 27312394 10.1016/j.wneu.2016.06.019

[7] Kalani M Couldwell W Extreme lateral supracerebellar infratentorial approach to the lateral midbrain J Neurol Surg Part B Skull Base 79 S415 S417 2018 10.1055/s-0038-1669981 PMC6240419 30456047

[8] Zaidi HA Mooney MA Levitt MR Dru AB Abla AA Spetzler RF Impact of timing of intervention among 397 consecutively treated brainstem cavernous malformations Neurosurgery 81 620 626 2017 28184444 10.1093/neuros/nyw139

[9] Zwagerman NT Wang EW Shin SS Chang YF Fernandez-Miranda JC Snyderman CH Does lumbar drainage reduce postoperative cerebrospinal fluid leak after endoscopic endonasal skull base surgery? A prospective, randomized controlled trial J Neurosurg [epub ahead of print October 19, 2018. DOI: 10.3171/2018.4.JNS172447] 10.3171/2018.4.JNS172447 30485224

